# Pro-Apoptotic Kinase Levels in Cerebrospinal Fluid as Potential Future Biomarkers in Alzheimer’s Disease

**DOI:** 10.3389/fneur.2015.00168

**Published:** 2015-08-04

**Authors:** Claire Paquet, Julien Dumurgier, Jacques Hugon

**Affiliations:** ^1^INSERM UMR-S942, Centre Mémoire de Ressources et de Recherche (CMRR) Paris Nord Ile de France, Groupe Hospitalier Lariboisière Fernand-Widal Saint-Louis, AP-HP, Université Paris Diderot, Paris, France

**Keywords:** cerebrospinal fluid, pro-apoptotic kinase, PKR, GSK-3, JNK, Alzheimer’s disease

## Abstract

Alzheimer’s disease (AD) is characterized by the accumulation of Aβ peptides, hyperphosphorylated tau proteins, and neuronal loss in the brain of affected patients. The causes of neurodegeneration in AD are not clear, but apoptosis could be one of the cell death mechanisms. According to the amyloid hypothesis, abnormal aggregation of Aβ leads to altered kinase activities inducing tau phosphorylation and neuronal degeneration. Several studies have shown that pro-apoptotic kinases could be a link between Aβ and tau anomalies. Here, we present recent evidences from AD experimental models and human studies that three pro-apoptotic kinases (double-stranded RNA kinase (PKR), glycogen synthase kinase-3β, and C-Jun terminal kinase (JNK) could be implicated in AD physiopathology. These kinases are detectable in human fluids and the analysis of their levels could be used as potential surrogate markers to evaluate cell death and clinical prognosis. In addition to current biomarkers (Aβ_1–42_, tau, and phosphorylated tau), these new evaluations could bring about valuable information on potential innovative therapeutic targets to alter the clinical evolution.

## Introduction

Neuropathological lesions in Alzheimer’s disease (AD) include senile plaques, neurofibrillary tangles, and amyloid angiopathy leading to synaptic and neuronal degradations. Aβ is formed after the cleavage of amyloid precursor protein (APP) by β secretase (BACE1) and γ secretase (1). According to the amyloid cascade hypothesis (2), biochemical and genetic findings have suggested that Aβ accumulation can induce tau phosphorylation and aggregation, synaptic dysfunction, and neuronal alteration, responsible for clinical signs of dementia (3). The precise mechanisms of neuronal demise have not been fully elucidated; however, apoptosis has been one of the most analyzed mechanisms in previous reports. Apoptosis is a sequence of events leading to the activation of caspases and cell disintegration. It has been proposed as the predominant form of cell death in AD due to unbalanced actions between pro and anti-apoptotic proteins (4, 5). Increased expression of several pro-apoptotic kinases has been observed in AD brains and their cellular pathways could be linked to AD physiopathology (6–13).

Protein kinases represent one of the largest super families, and they are molecular switches activating and inhibiting many biological processes, such as memory, differentiation, cell division, and cell death. They belong to complex metabolisms interacting with other kinases and their dysfunctions can be associated with various diseases (14). According to Hardy’s hypothesis (3), Aβ peptides can trigger protein kinases in AD participating in neuronal signaling pathways between Aβ and tau phosphorylation. Many of these kinases are also pro-apoptotic and could induce synaptic and neuronal sheddings.

Recently, reports have shown that cerebrospinal fluid (CSF) levels of Aβ_1–42_, total tau (T-tau), and phosphorylated tau (ptau) were altered in AD patients and in patients with mild cognitive impairment (MCI) with higher risks to convert to AD (15). The analysis of CSF biomarkers has brought about new insights into the managing procedures of AD patients leading to new diagnostic criteria (16). The levels of these CSF biomarkers correlate with the severity of neuropathological lesions (17, 18). The use of CSF biomarkers has improved the confidence of clinicians for AD diagnosis (19) and now serves for the screening of patients in clinical trials (20). Because lumbar puncture (LP) is better known, more practiced, and well tolerated (21–24), physicians resort in CSF biomarkers more and more in clinical practice reflecting the impact on AD diagnosis (25, 26).

However, these classic CSF biomarkers are not directly predictive of the AD evolution. The need for new biomarkers remains to avoid the classification of patients by quintiles or clusters (27, 28). Furthermore, they have several pre-analytical requirements limiting the analysis to expert centers while AD patients are located everywhere in the world (29–31). Consequently, additional biomarkers are needed to predict the clinical evolution and cognitive decline, and to assess the efficiency of treatment targeting pathways of neuronal death.

This brief report will provide an overview of three pro-apoptotic protein kinases that are involved in AD physiopathology with detectable levels in biological fluids. We aim to address their place as new biomarkers reflecting the rate of neuronal death, predicting possibly the clinical evolution and requiring less pre-analytical preparations.

## Pro-Apoptotic Kinases in Alzheimer’s Disease

### Double-stranded RNA-dependent protein kinase

#### Involvement of PKR in AD Pathophysiology

RNA-dependent protein kinase (PKR) is a serine/threonine pro-apoptotic kinase present in cells as non-activated. PKR plays a role in various cell functions (32) and is involved in apoptosis (33–40). Activation of PKR results from autophosphorylation on threonine residues 446 and 451 of the kinase domain (41–45). Once activated, PKR triggers several effectors and pathways leading to apoptosis including the activation of eukaryotic initiating factor 2 alpha (eiF2α), which inhibits protein synthesis. PKR participates in the activation of caspase 8, which can contribute to the conversion of procaspase 3 into caspase 3 (46, 47). More widely, PKR activates both intrinsic and extrinsic apoptotic pathways (38, 48–51). Activated and pro-apoptotic forms of PKR (pPKR) can accumulate in several neurodegenerative diseases including AD (9, 12, 52–54). In 2002, we have observed an abnormal activation of PKR in AD brains (9) and this result was confirmed by several teams (12, 52, 53). Immunohistochemical findings performed in AD brains revealed an accumulation of pPKR around senile plaques (in dystrophic neurites), in the cytoplasm of neurons especially in the hippocampus and the temporal cortex, whereas neuronal staining was more nuclear in the frontal and parietal cortex (8, 55, 56).

Using animal and cells models, we have shown that PKR is activated by Aβ peptide (8, 53, 55–61) through its activator PACT (56), and this activation plays a role in neuronal death in AD (8, 55, 56), Furthermore, we have shown that the activation of PKR (partly by Aβ) could control the levels of β-secretase (BACE1) in stressed cells. These data suggest the existence of a pathological self-sustaining loop involving PKR (1). On the other hand, we have reported a co-localization of ptau and pPKR (8). In neural cell cultures, Aβ induced the phosphorylation of PKR, glycogen synthase kinase-3β (GSK-3β), and tau. The pharmacological inhibition of PKR reduced GSK-3 activation and tau phosphorylation, suggesting that PKR could indirectly control the abnormal formation of tangles (8). Moreover, PKR has been shown to be implicated in memory (62). All these findings suggest that PKR plays a key role in the events leading to abnormal molecular signals at the origin of neurodegeneration in AD.

#### PKR as a New Biomarker

The analysis of PKR levels in biological fluids of AD patients was further explored. In 2006, we observed increased pPKR levels in lymphocytes from AD patients compared to controls. However, an important overlap between the two groups was found showing that this biological test was not appropriate for diagnosis (63). The CSF is in direct contact with the brain and is less influenced by peripheral factors. We have evaluated CSF PKR concentrations in AD, MCI, and control individuals. Ninety one patients were included. The levels of total PKR (T-PKR), pPKR, Aβ, T-tau, and ptau were determined by Western blots or ELISA methods. The concentrations of T-PKR and pPKR were significantly increased in AD patients and in most MCI patients compared to neurological controls. The optimal threshold for pPKR to discriminate AD patients from controls gave a sensitivity of 91.1% and a specificity of 94.3%. In the group of AD patients, concentrations of pPKR correlated with CSF levels of tau and ptau. A few AD or MCI patients with normal Aβ and tau levels had increased pPKR levels. A correct discrimination between non-AD subjects and AD patients was possible with CSF PKR evaluations (11). To understand if pPKR and ptau could be found in extracellular fluid after the induction of neural endoplasmic reticulum (ER) stress, we have carried out a study in the supernatant of stressed human neuroblastoma cells. Results have revealed an increased concentration of T-PKR, pPKR, T-tau, and ptau after cellular stress. pPKR, together with tau, are released from neural cells due to an increased membrane permeability of unknown origin or late breakdown of the apoptotic plasma membrane. The fact that especially pPKR is increased in AD CSF (317%) and much less T-PKR (38%) could suggest that mainly pPKR accumulates in affected neurons before being released in the extracellular space of AD brains (11).

In a second step, we have analyzed in the same cohort, the predictive value of CSF pPKR levels on the cognitive decline over 2 years (11, 64). Every 6 months, patients underwent neurological exams and neuropsychological assessments including a Mini Mental State Examination (MMSE) evaluation. Using a multivariate linear mixed model, our results showed that the level of CSF pPKR was associated with a more pronounced cognitive decline. In this cohort, CSF pPKR levels were the only biomarkers linked to the cognitive decline over the follow-up survey. Furthermore, although classical biomarkers are very useful to predict the clinical outcome of MCI patients, the results of biomarker levels in the two groups of amnestic MCI patients (converters and non-converters) show that PKR was the most discriminant biomarker between the two groups (64).

### Glycogen synthase kinase-3 protein kinase

#### GSK-3β and AD Pathophysiology

Glycogen synthase kinase-3 (GSK-3) is a proline-directed serine/threonine kinase and is ubiquitously expressed with two isoforms, GSK-3α and GSK-3β (65, 66). GSK-3 has a role in many biological pathways including gene transcription, apoptosis (67), regulation of glycogen metabolism (68, 69), and microtubule stability (70, 71). GSK-3β is highly present in neurons (72) and can phosphorylate tau at 17 sites of the protein, more extensively than any other kinases (71). It is activated on two phosphorylation sites (Tyrosine 216 and Serine 9), which have opposite effects. Tyr216 phosphorylation leads to GSK-3β activation while serine 9 phosphorylation inhibits its activity (66). Evidences from several works have suggested that the involvement of GSK-3 in AD is linked to the reduction of acetylcholine synthesis (66) and to increased production of Aβ. GSK-3β can co-localize with ptau in dystrophic neurites and tangles (8, 73, 74). Enhanced GSK-3 protein levels and activity were observed in the frontal cortex and hippocampus in AD brains (8). Furthermore, in 2012, we have shown that pPKR, activated GSK-3β, and ptau proteins can be co-expressed in AD brains. In addition, PKR can modulate neuronal apoptosis and tau phosphorylation through GSK-3β activation. GSK-3 inhibition decreased tau phosphorylation without acting on PKR activation (8). It has recently been reported that a polymorphism in the GSK-3 promoter region is a risk factor for late onset AD (75). We have also shown that active Aβ immunotherapy in AD patients induced a reduction of all GSK-3β forms; active, inactive, and total (76). Overall, the inactive GSK-3β appears to be the more abundant form compared to the active form. Finally, GSK-3 is pro-apoptotic and thereby might directly contribute to neuronal death in AD (67).

#### GSK-3 as a Biomarker

In 2004, Hye et al. explored GSK-3 levels in circulating lymphocytes. Total GSK-3α and β and inactive GSK-3β concentrations were assessed in white blood cells in a series of 113 patients including AD, MCI, and elderly controls. The results showed increased GSK-3α (+65%) and GSK-3β (+59%) protein levels in AD and MCI compared to controls without concomitant augmentation of pGSK-3β (77).

In 2004, a decreased level of CSF GSK-3β in schizophrenic patients from a small cohort has been shown (78). The study did not evaluate activated or inactivated form of the kinase and so far no study has assessed CSF GSK-3 concentrations in AD patients.

Taking together, measurements of GSK-3 in biological fluids could be a supplemental biomarker reflecting AD pathology. Since GSK-3 is dramatically decreased in AD brains after active Aβ immunization (76), CSF GSK-3 evaluation could reflect the efficiency of this therapeutic on neuronal stress and pro-apoptotic pathways.

### C-Jun N-terminal kinases

#### JNK and AD Pathophysiology

C-Jun N-terminal kinases (JNKs) are a family of serine/threonine protein kinases encoded by three genes (JNK1, JNK2, and JNK3). JNK1 and JNK2 are ubiquitous, and JNK3 is mainly expressed in the brain. JNKs are activated by phosphorylation (pJNK) through mitogen-activated protein (MAP) kinase kinase pathways induced by extracellular stimuli, such as cytokines and Aβ peptides (79). JNKs have multiple functions, including regulation of gene expression, inflammation, cell proliferation, and apoptosis (80). In the brain, while JNK1 and 2 are involved in the development, JNK3 seems principally implicated in neurodegeneration (81). Previous studies have revealed that JNKs, particularly JNK3, can control BACE1 expression levels (82), can phosphorylate APP, and enhance Aβ production (83, 84). The deletion of JNK3 has a neuroprotective effect against ischemia (85) and excitotoxicity (86, 87). Aβ-induced cell death is reduced in cultures of cortical neurons from JNK3 knockout (KO) mice, and JNKs have been implicated in experimental models of AD and Parkinson’s disease. pJNK is increased in AD brains as well as upstream JNKs activators (88).

Immunohistochemical findings in AD brains have shown that the activated form of JNK (pJNK) was localized in peripheral rims of senile plaques, in neurofibrillary tangles, and granulovacuolar degenerations, as previously reported (7, 88). Neurons were modestly marked in the cytoplasm and in the nucleus in AD brains. In control brains, pJNK immunolabellings were rarely detected. The full form of JNK3 was detected, in the center and around senile plaques, as well as in the cytoplasm of neurons. Confocal imaging revealed an association between Aβ42 and JNK3 stainings in senile plaques, suggesting that JNK3 proteins may accumulate during the formation of amyloid aggregates (7). Immunochemical results revealed a significant correlation between Aβ and JNK3 levels in control and AD brains. In frontal cortex, pJNK and JNK3 could be detected in the same senile plaques. Quantification of these histological results showed an increase of JNK3 staining (+59%) and pJNK staining (+182%) in AD brains compared to control brains (7).

#### JNK as a CSF Biomarker

According to these results, JNK3 could be a marker of abnormal pathways in the CSF. In a recent study, CSF JNKs levels were evaluated by western blots in AD patients and neurological controls. JNK1, JNK2, and pJNK proteins were not detectable in the CSF. A significant increase of CSF JNK3 levels was found in AD patients compared to controls (+23%). Optimal cut-offs showed that the JNK3 value of 70.3 optical density units (ODU) had a sensitivity of 80% and a specificity of 73%, with an area under curve of 0.75 (7). No correlations were found between CSF JNK3 levels and age, sex, CSF levels of Aβ_1–42_, T-tau, and pTau, as well as MRI evaluations using Fazekas scores (89) or Scheltens scales (90).

Patients with AD were followed for a mean period of 1.8 (±1.3) years. During the follow-up, clinicians performed 4.7 (±2.25) MMSE tests per patient. Using linear mixed models, a longitudinal analysis using tertiles of JNK3 levels was carried out. We found that patients in the third tertile (>89 ODU) experienced a reduced and significant decline in MMSE scores over time. This association was maintained after adjusting for age, sex, educational levels, and MRI abnormalities. Comparison of the tertiles revealed that patients in the two lowest tertiles (<89 ODU) experienced a more rapid decline of MMSE scores over time than those in the upper tertile (7).

## Conclusion

Apoptosis seems an important way of cellular death in AD. Several pro-apoptotic kinases are involved in the pathophysiology of AD, and the evaluations of CSF concentrations could be useful to predict the cognitive decline. In addition, since these three kinases are implicated in neuronal apoptosis, they represent new therapeutic targets that could afford neuroprotection and alter the relentless clinical evolution in AD patients.

## Conflict of Interest Statement

Claire Paquet, Julien Dumurgier, and Jacques Hugon are investigators in Aβ immunotherapy trials for Lilly and Eisai. Jacques Hugon is a consultant for Roche in relation to Aβ immunotherapy trials. In our part, studies on PKR were funded by French Alzheimer Plan, studies on GSK-3β were funded by NEURAD (Marie Curie funding), studies on JNK received University and INSERM funding. The funders had no role in study design, data collection and analysis, decision to publish, or preparation of the manuscript. Patent and license: CSF PKR levels received a world patented and license « Méthodes de diagnostic des maladies neurodégénératives »(BE11754519.4 PCT/IB2011/053571).
